# Menstrual Dysfunction in Adolescent Female Athletes

**DOI:** 10.3390/sports12090245

**Published:** 2024-09-04

**Authors:** Valeria Calcaterra, Matteo Vandoni, Alice Bianchi, Agnese Pirazzi, Lara Tiranini, Paola Baldassarre, Marianna Diotti, Caterina Cavallo, Rossella Elena Nappi, Gianvincenzo Zuccotti

**Affiliations:** 1 Department of Internal Medicine and Therapeutics, University of Pavia, 27100 Pavia, Italy; 2 Pediatric Department, Buzzi Children’s Hospital, 20154 Milano, Italy; alice.bianchi1@unimi.it (A.B.); paola.baldassarre@unimi.it (P.B.); marianna.diotti@gmail.com (M.D.); gianvincenzo.zuccotti@unimi.it (G.Z.); 3 Laboratory of Adapted Motor Activity (LAMA), Department of Public Health, Experimental Medicine and Forensic Science, University of Pavia, 27100 Pavia, Italy; matteo.vandoni@unipv.it (M.V.); agnese.pirazzi01@universitadipavia.it (A.P.); caterina.cavallo01@universitadipavia.it (C.C.); 4 Department of Clinical, Surgical, Diagnostic and Pediatric Sciences, University of Pavia, 27100 Pavia, Italy; lara.tiranini01@universitadipavia.it (L.T.); nappi@rossellanappi.com (R.E.N.); 5 Research Center for Reproductive Medicine, Gynecological Endocrinology and Menopause, Fondazione IRCCS Policlinico San Matteo, 27100 Pavia, Italy; 6 Department of Biomedical and Clinical Sciences, University of Milano, 20157 Milano, Italy

**Keywords:** menstrual dysfunction, menstrual disorders, menstrual cycle, physical exercise, stress, young females, female athletes

## Abstract

Despite the benefits of exercise on mental and physical health, excessive training loads can lead to health problems in the long term, including a wide spectrum of menstrual dysfunction (MD). This narrative review aims to analyze the relationship between physical exercise and MD in adolescent female athletes to support regular menstrual health monitoring and promote educational programs on reproductive risks. When dealing with MD in young athletes, several factors entangled with maturation of the hypothalamus–pituitary–ovarian axis should be considered. Firstly, some disciplines seem to have a higher prevalence of MD due to the high loads of training regimes and the early introduction of athletes to a competitive career. Moreover, low energy intake and a low body mass index appear to exacerbate existing MD. Lastly, disordered eating behaviors and psychological stress can contribute to MD in female athletes. The type of sport, influencing the intensity and duration of exercise, as well as individual psycho-physiological and environmental factors, may influence the role of physical activity in the manifestation of MD. Early recognition and management of MD, along with collaboration between sports organizations and health professionals, are crucial to minimize risks, ensure proper nutrition, and balance training with recovery. Keeping an open discussion on the topic may prospectively improve awareness, early diagnosis, and treatment strategies, as well as reduce injury risk and enhance sports performance.

## 1. Introduction

Adolescence is a peculiar stage of life characterized by key physical, psychological, cognitive, and behavioral changes that lay the foundation for adulthood. In girls, many neuroendocrine, hormonal, and metabolic factors contribute to the pubertal activation of the hypothalamic–pituitary–ovarian (HPO) axis, leading to the development of secondary sexual characteristics and the occurrence of menarche followed by periodic menstruation [1]. A healthy lifestyle during childhood and adolescence is crucial to promote hormonal balance and regular ovulatory and menstrual function, while unhealthy habits are associated with altered timing of puberty and menstrual dysfunction (MD) in adolescents and adults [2,3].

According to the World Health Organization (WHO), physical activity in young people is known to provide multiple health benefits in the cardiometabolic, respiratory, muscular, and bone systems [4]. Despite that, a recent global survey revealed that 84.7% of girls aged 11–17 years are insufficiently physically active [5], thus becoming at risk of overweight and obesity with a detrimental effect on reproductive function [6]. At the same time, excessive energy expenditure with physical activity is a well-known risk factor for MD, especially functional hypothalamic amenorrhea (FHA), a condition of hypogonadotropic hypogonadism due to low energy availability and/or high levels of stress [3,7]. The mechanisms through which exercise can affect the menstrual cycle depend on several factors, such as the type of sport, specific training requirements, intensity, frequency, and duration of exercise [8,9], with the highest rates of FHA in esthetic, endurance, and weight-class sports [7]. In addition to exercise, eating disorders also play a significant role in energy availability. Indeed, a recent meta-analysis revealed a higher prevalence of disordered eating behaviors (i.e., drive for thinness, restricting, loss-of-control eating) in athletes participating in esthetic/lean sports compared to non-athletes [10]. In the context of overtraining and/or eating disorders, Relative Energy Deficiency in Sports (RED-S) is a condition of low energy availability that significantly impairs general and reproductive health and sport performance [11,12]. Low energy availability, in fact, compromises gonadotropin-releasing hormone (GnRH) secretion, reducing luteinizing hormone (LH) concentration and normal follicle-stimulating hormone (FSH) secretion. Thus, the balance between energy expenditure and energy intake must be considered in exercising adolescents and young women for its significant impact on menstrual function and fertility. Finally, psychological stress and performance anxiety in competitive sports may also alter the neuroendocrine substrates of the HPO axis, potentially contributing to MD and amenorrhea [7,13]. Exercise-induced physical stress contributes to increased levels of cortisol in the blood, which can chronically alter LH pulsatility and estrogen levels [8]. Moreover, weight loss linked to intense exercise can further reduce estrogen levels, as adipose tissue is a key site for estrogen production [14]. 

Encouraging physical activity in adolescence is fundamental for its known benefits. Nonetheless, it is equally important to increase the awareness of the impact of energy deficiency on women’s health [12].

The aim of the present narrative review is to analyze the relationship between physical exercise and MD, with a focus on adolescent female and young athletes, in order to support regular monitoring of menstrual health and promote educational programs about reproductive risks over time. Indeed, girls may experience irregular and anovulatory cycles during the first and second years after menarche due to an immature HPO axis, but a timely MD diagnosis in adolescent athletes is essential to implement personalized strategies for balancing proper nutrition and training, thereby protecting their health.

## 2. Methods

We conducted a narrative review [15] to explore the impact of physical exercise on MD in young female athletes. An extensive literature search was performed using the electronic database PubMed. The search included English-language studies involving females under 21 years of age, to target adolescents and young females. Regarding types of manuscripts, original research articles, systematic and narrative reviews, meta-analyses, and longitudinal studies were considered. Case reports, case series and letters, and all non-English-language manuscripts were excluded. The following search terms (alone and/or in combination) were employed: physical exercise, sports, young females, adolescents, female athletes, menstrual disorders, menstrual dysfunction, menstrual cycle. Initially, a large number of articles were considered (*n* = 720). These were refined by screening the abstracts (*n* = 148) and performing detailed full-text evaluations of relevant studies (*n* = 84), during which key findings, inclusion criteria (age and sex of enrolled subjects, all sports, all types of menstrual disorders), and study characteristics (type of article, manuscript language) defined as research criteria were respected. These studies were critically analyzed for inclusion in the manuscript (*n* = 56). Additionally, the reference list of all articles was checked to identify relevant studies.

**Figure 1:**
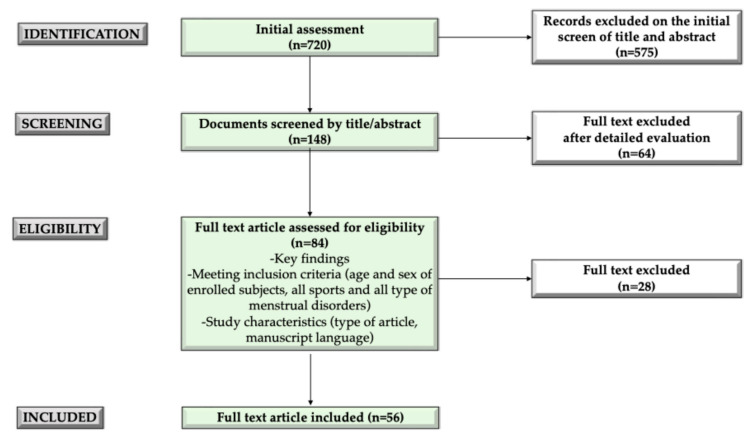
The manuscript selection process.

In Figure 1, the selection process of the manuscript is reported.

## 3. Menstrual Cycle and Menstrual Dysfunction in Adolescents

### 3.1. The Normal Menstrual Cycle

Menarche, defined as the first menstrual bleeding, typically occurs within 2 to 3 years after thelarche (breast development) at Tanner stage IV. In well-nourished populations, the median age of menarche is 12–13 years and is 12.5 among all ethnicities [16,17,18,19]. A lower body mass index (BMI) can be associated with delayed menarche [20,21]. Research indicates that approximately 17% body fat is necessary for menarche, and 22% is required to maintain regular menses in older adolescents [22]. 

The normal menstrual cycle length in adolescents ranges from 21 to 45 days, with an average of 34.5 days. The duration of menstrual bleeding lasts between 2 and 7 days, with most girls menstruating for 4 to 5 days. The first cycle is typically the longest [16,23]. In the first and second year after menarche, girls experience irregular and anovulatory cycles due to the immaturity of the HPO [16,18,22,24], but a period of amenorrhea (absence of menstruation) lasting more than three months or 90 days is infrequent and should be medically evaluated [16,22]. In the years following menarche, menstrual cycles gradually shorten and the frequency of ovulatory cycles increases [23]. By three years post menarche, most girls have cycles that resemble those of adults, typically ranging from 21 to 34 days [16,22,23]. Normal menstrual flow involves the use of three to six pads or tampons daily, with an average blood loss of 30–40 mL [19,20]. Dysmenorrhea, or painful menstruation, is common in adolescents, with a prevalence similar to that in adults [23]. 

The menstrual cycle is a meticulously regulated process orchestrated by the HPO axis through a complex system of hormonal feedback. The cycle can be divided into three distinct phases: follicular (proliferative), ovulatory, and luteal (secretive). The follicular phase starts with the onset of the menses and ends with ovulation. It is the most variable phase in terms of length, ranging from 7 to 22 days. During the early follicular phase, low levels of estradiol and progesterone stimulate the release of GnRH by the hypothalamus. GnRH acts on the pituitary gland, prompting the secretion of FSH and LH. The increase in FSH leads to the recruitment of a cohort of primordial follicles in the ovaries. One follicle reaches full maturation and is selected as the dominant follicle. LH stimulates ovarian theca cells to produce androgens, while FSH stimulates the ovarian granulosa cells to convert androgens into estrogens via the enzyme aromatase. The dominant follicle produces high quantities of estradiol, which promotes the proliferation of the endometrial lining. Increasing estradiol levels suppress FSH and LH production through negative feedback on the pituitary gland. Eventually, a switch from negative to positive feedback driven by estradiol occurs, leading to a surge of LH levels (mid-cycle). This LH surge is the trigger for ovulation: the dominant follicle disrupts and releases an oocyte. This oocyte resumes meiosis and begins its journey through the fallopian tube, preparing for potential fertilization. The luteal phase follows ovulation and typically lasts about 14 days, marking a more constant duration compared to the follicular phase. The high LH levels transform the ruptured follicle into the corpus luteum, which starts to produce significant amounts of progesterone, a crucial hormone for transitioning the endometrium from the proliferative phase to the secretory phase, preparing the uterus for embryo implantation. If fertilization does not occur, the corpus luteum degenerates into the corpus albicans and progesterone levels decrease, causing constriction of the arterioles and shedding of the endometrial lining through menstruation. The cycle then restarts with low levels of estradiol and progesterone stimulating the release of GnRH [17,22,25]. 

In adolescents, estradiol sometimes fails to induce the (LH) surge consistently, resulting in the absence of ovulation. Without ovulation, the dominant follicle degenerates, and in the absence of a luteal corpus, progesterone is not produced. Consequently, the endometrium continues to proliferate under the influence of estradiol alone. This prolonged proliferation leads to irregular shedding of the endometrium, causing abnormal bleeding patterns during the peri-menarchal period [20].

### 3.2. Menstrual Dysfunction in Adolescents

The menstrual cycle is determined by a synchronic response involving the HPO axis, and its delicate equilibrium can easily be altered by biological and psychological factors. 

Despite individual variations, normal menstrual cycles occur in adults with a frequency between 25 and 37 days and last less than 8 days [26]. Instead, irregular menstrual cycles are considered normal during adolescence in the first year post menarche, while a menstrual frequency between 21 and 41 days is regular until 3 years post menarche. Menstrual cycle abnormalities in adolescents may also have an organic cause, but diagnosis is often delayed due to a lack of awareness of what it is normal in terms of bleeding pattern [27]. 

**Figure 2:**
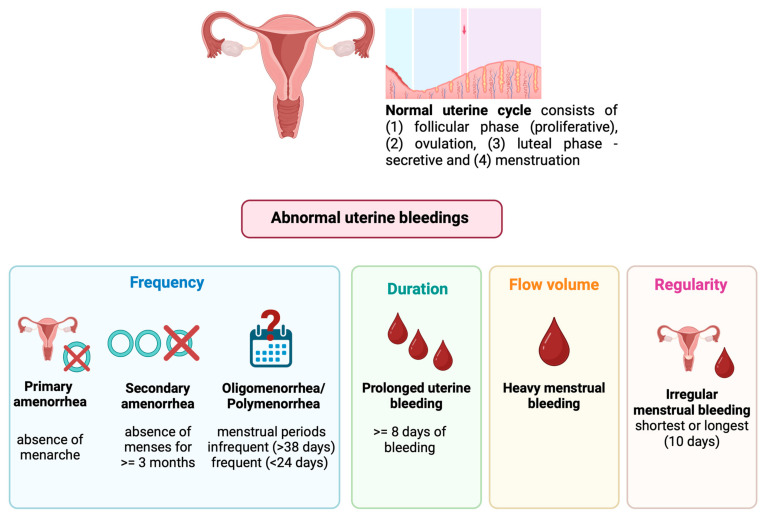
Abnormal uterine bleedings according to the International Federation of Gynecology and Obstetrics [27]. Created by Biorender^®^.

MD encompasses both ovulatory disorders and abnormal uterine bleedings (AUBs) according to the International Federation of Gynecology and Obstetrics (FIGO) [26,27]. As illustrated in Figure 2, applying the FIGO System in a clinical setting requires a structured history that captures four parameters: frequency, duration, regularity, and subjective flow volume.

Abnormal frequency in adults may manifest as oligomenorrhea/infrequent menstrual periods (>38 days), polymenorrhea/frequent menstrual periods (<24 days), and amenorrhea (primary or secondary) [26,27]. Abnormal duration causes prolonged uterine bleeding (>8 days). Abnormal flow volume can cause heavy menstrual bleeding (HMB). Intermenstrual bleeding or unscheduled bleeding under hormonal therapy refer to irregular bleedings outside menstrual flow. Finally, the menstrual cycle is defined as irregular if periods have a variation between each other over 8–10 days [26,27]. 

Primary amenorrhea is defined as the absence of menses at age 15 years in the presence of normal growth and secondary sexual characteristics or at age 13 years in the complete absence of secondary sexual characteristics [28]. Furthermore, evaluation for primary amenorrhea should be considered in the absence of menses 3 years after thelarche or, alternatively, 5 years, if thelarche occurred before the age of 10 years [28].

Secondary amenorrhea is defined as the absence of menses for 3 or more months in women with previous regular menses, or for 6 months in women with previously irregular menses. The prevalence in the general population is around 5–12.2% [29].

The different causes of amenorrhea and other menstrual abnormalities are reported in Table 1.

Oligomenorrhea is characterized by infrequent menstrual periods, i.e., more than 45 days within 3 years post menarche and more than 38 days after 3 years from menarche. Instead, polymenorrhea consists of frequent menstrual periods, i.e., less than 21 days within 3 years post menarche and less than 24 days after 3 years from menarche. The main causes of altered frequency of the menstrual cycle include endocrine disorders (thyroid dysfunction, hyperprolactinemia, hyperandrogenism, hypothalamus–pituitary axis disorder), polycystic ovary syndrome (PCOS), and ovarian insufficiency (Table 1). According to the literature, the prevalence of oligomenorrhea in the general population ranges from 6 to 15.3% [29].

HMB is defined as more than 8 days of bleeding, greater than 80 mL of blood loss per cycle, or using more than seven pads a day. An interesting approach for the assessment of menstrual blood loss is PBAC (Pictorial Blood Loss Assessment Chart) score. It is a semi-quantitative scoring system which considers the number of sanitary products used, the degree to which these products are soiled with blood, the number and size of blood clots passed, and the number of flooding episodes [30]. A PBAC score > 100 is strongly related to HMB. The most common etiology of HMB is an immature HPO axis, but bleeding abnormalities as coagulation defects, thrombocytopenia, and platelet dysfunctions should also be considered (Table 1) [31].

### 3.3. The Evaluation of Menstrual Dysfunction

The evaluation of menstrual abnormalities begins with a comprehensive investigation of the patient’s full medical history: lifestyle (diet, physical activity, habits); chronic illness, sexual history, medication exposure, family history (bleeding disorders, autoimmune disorders, menstrual history from first-degree relatives); and personal menstrual history (onset of pubertal development, age of menarche, frequency and length of menstrual cycle, quantity and color of blood flow, and associated symptoms). A detailed physical examination should be performed, including anthropometric measurements, skin inspection, visual field testing, thyroid palpation, evaluation of secondary sexual characteristics, and abdominal and pelvic palpation. External genital examination should always be conducted, while speculum examination is doable in sexually active women to visualize the cervix and vaginal canal. Laboratory evaluation needs to include a human chorionic gonadotropin test to rule out pregnancy, a complete blood cell count, and standard coagulation testing. Additional testing is based on clinical suspicion: sexually transmitted infection (STI) testing in sexually active adolescents; thyroid function tests to rule out thyroid disorders; androgens and SHBG tests in patients with signs and symptoms of PCOS; prolactin levels test to check for hyperprolactinemia; FSH, LH, and estradiol measures to basically explore the HPO axis; and a Von Willebrand panel (vWF antigen level, ristocetin cofactor activity, and factor VIII activity) if a bleeding disorder is suspected [17,19,20,32]. Routine imaging is generally not indicated for AUB in adolescents due to the rarity of structural causes. However, imaging should be considered based on clinical judgment: pelvic ultrasonography is the most reliable modality. When transvaginal ultrasonography is not appropriate or sufficient, magnetic resonance provides a more sensitive view of pelvic anatomy [19,20].

## 4. The Interplay between Exercise and the Menstrual Cycle

Compared to non-exercising young women, professional athletes gain numerous benefits, including reduced substance abuse risk, improved self-esteem, better academic performance, and lower incidences of depression and chronic disease. Despite the benefits of exercise on mental and physical health, excessive training loads can lead to health problems in the long term, including a wide spectrum of MD. 

The interplay between exercise and the menstrual cycle has been extensively studied, revealing a spectrum of effects ranging from beneficial to potentially disruptive. 

MD may be related to anovulation that occurs because of an immature HPO axis or low energy availability, both typical conditions of young athletes [33].

FHA is a condition of chronic anovulation not due to any organic cause, but attributable to weight loss, exercise, or stressors [7]. In the pathophysiology of FHA, energy deficiency and/or stressful situations are the key determinants of a neuroendocrine adaptation characterized by functional reduction in the GnRH drive. This results in reduced LH pulse frequency and low FSH levels that cause subsequent hypoestrogenism [7,34]. Chronic hypoestrogenism is of major importance for its long-term health consequences, such as infertility, deficiency in bone mineral density, cardiovascular diseases (endothelial dysfunction, dyslipidemia), cognitive impairment, psychiatric disorders (depression, anxiety), and increased mortality for all causes [34,35]. Thus, FHA should always be considered in exercising adolescents or young women with low energy availability.

Energy imbalance caused by restricted nourishment and/or excessive energy expenditure influences the HPO axis through various metabolic signals that act on the neuroendocrine regulation of GnRH release [7]. Also, the altered body composition with reduced body fat and increased muscle mass associated with intense training modifies hormone production from adipose tissue and activates endocrine adaptive mechanisms aimed to safeguard energy for vital functions [7]. Leptin is an anorexigenic peptide secreted by adipocytes that mediates information about energy availability. Leptin signaling acts on kisspeptin neurons, and low leptin levels found in low-energy conditions, FHA, and athletes have been associated with GnRH suppression [36]. Energy-deficient states, including FHA, are associated as well with low insulin, an anabolic hormone produced by pancreatic beta cells to regulate glucose homeostasis [37]. In addition, when exploring the role of thyroid function in amenorrheic athletes, studies revealed low levels of total triiodothyronine (T3) as a mechanism to decrease the resting metabolic rate and favor energy preservation [38]. Other metabolic mediators are increased in energy imbalance conditions. Adiponectin is an adipokine produced by adipose tissue. It rises with intense physical activity associated with weight loss and decreased body fat, and high levels of adiponectin influence the HPO axis through GnRH suppression and altered kisspeptin gene transcription [39]. Even elevated ghrelin inhibits reproductive function through an altered hypothalamic expression of kisspeptin genes [40]. Ghrelin is an orexigenic peptide secreted by gastric cells whose levels are high in women with amenorrhea, eating disorders, low body weight, and low fat mass [41]. Peptide-YY is secreted by the distal gut in response to food intake to induce satiety and is found to be elevated in amenorrheic athletes when compared to non-amenorrheic athletes [42]. Irisin is a recently discovered hormone with pleiotropic actions, mainly on metabolism and bone health. It is secreted principally by the skeletal muscle during physical activity and secondly by white adipose tissue, and it mediates the crosstalk between muscle and other tissues [43]. Some evidence suggests a direct influence of altered levels of irisin on GnRH and gonadotropin secretion [43]. Indeed, lower levels of irisin are observed in young amenorrheic athletes [44] and in women with psychogenic FHA [45], raising concerns for the detrimental effect of irisin depletion on bone metabolism that adds up to hypoestrogenic-induced bone loss [43].

In addition to low energy availability and body composition imbalance, high levels of psychosocial stress and performance anxiety can affect the female reproductive system. Stress responses are mediated by the hypothalamus–pituitary–adrenal (HPA) axis, in which the hypothalamic corticotropin-releasing hormone (CRH) stimulates pituitary secretion of adrenocorticotropic hormone (ACTH) that in turn triggers cortisol release from adrenal glands [40]. The HPA axis interferes with the HPO axis through CRH, which acts on kisspeptin neurons exerting a final inhibitory effect on GnRH pulses. Elevated cortisol also suppresses the release of GnRH and gonadotropins [40]. Athletes and women with FHA manifest a chronic activation of the HPA axis with a potential detrimental effect on reproductive function. Indeed, in addition to common psychogenic stressors, athletes might be exposed to other sources of stress, such as performance pressure, strict training, intense competition, anxiety, coping with injuries, and interpersonal conflicts within the athletic team, that maintain a hyperactivated HPA axis when individual resilience to allostatic load is low [46].

Energy imbalance and psychological stress may increase the risk of developing athlete triad syndrome [47]. This syndrome is typical of physically active women and is characterized by the association of the following conditions that impair general health: (i) low energy availability (with or without eating disorders), (ii) menstrual cycle disturbances, and (iii) low bone mineral density [33]. In addition, adolescence is a decisive stage during which most of body’s energies are employed in bone accrual, growth, and development; therefore, the diagnosis of exercise-associated MD is of primary concern [48].

The impact of physical activity on menstrual function may be influenced by the type, intensity, and duration of exercise, as well as individual psycho-physiological and environmental factors.

Low energy availability associated with MD can be considered an alarm bell indicating worsened health status and decreased performance levels. Generally, moderate physical activity offers advantages such as maintaining a healthy body weight, reducing cardiovascular disease and diabetes risks, and positively influencing reproductive function and breast cancer risk. On the contrary, high-intensity exercise, especially when coupled with low energy availability, can lead to MD [8].

It is essential to understand these effects to promote physical activity guidelines that enhance overall health without compromising menstrual function. Early identification, appropriate management, and continuous monitoring of MD in female athletes are essential to safeguard their menstrual health and optimize sports performance. Coaches, trainers, and physicians should monitor menstrual cycle regularity in athletes, particularly in high-risk sports, as MD can have an impact on overall health. 

## 5. Menstrual Dysfunction in Adolescent Female Athletes

In recent years, the popularity of competitive sports and the participation of women in sports and top-level competitions have substantially increased, especially among the youth. Young female athletes have then adapted their usual training regimes to match and fulfill the higher performance standards of elite competitions, with increases in the intensity, frequency, and volume of their physical training [47].

### 5.1. Menstrual Dysfunction across Sports

Gimunová et al. [29] highlight the prevalence of MD in female athletes across various sports, identifying associated risk factors and how variables like sport type, training intensity, and nutritional status influence menstrual patterns. Overall, reproductive abnormalities are observed in a wide range from 6 to 79% of exercising women. Young female athletes are often exposed to delayed puberty, MD (oligomenorrhea, amenorrhea, HMB), and premenstrual syndrome symptoms [1]. MD is more frequent in sports like gymnastics and endurance disciplines, with significant rates also seen in team sports such as volleyball and soccer compared to the general population [49,50]. Primary amenorrhea occurs in less than 1% of the general population, but it is significantly higher in athletes, reaching 53.8% in rhythmic gymnasts, 20% in soccer players, and 19% in swimmers [8,51]. Secondary amenorrhea is highly prevalent in sports such as cycling (56%), triathlon (40%), and rhythmic gymnastics (31%) [29,52,53]. Oligomenorrhea has a general population prevalence of 6–15.3%, but higher rates are found in sports like boxing (55%), rhythmic gymnastics (44%), and artistic gymnastics (32%).

Coelho et al. [54] in their cross-sectional study found that young tennis athletes suffered from menstrual irregularity due to the low energy availability caused by eating disorders associated with exercise stress [54]. Similarly, De Souza et al. [55] showed that menstrual and hormonal disturbances move on a continuum varying accordingly to the degree of energy deficit; also, Gibbs et al. [56] recently outlined the association between tendencies to follow a hypocaloric diet, a psychometric indicator of an eating disorder, with higher frequency of MD in exercising women.

Moreover, MD leads to low bone mineral density and delayed development of secondary sexual characteristics during puberty [57]. For example, the prevalence of low bone mass in relation to MD was observed in a sample of adolescent endurance runners [58,59], and similar findings were reported by Beals et al. [60] in their study on collegiate runners. Variables associated with lower bone mass in the adolescent runners included higher levels of training, menstrual irregularity, and restrained dietary intake [61]. In fact, the absence of a physiological balance during sport practice may lead to alterations in gonadotropins, androgens, estrogens, progesterone, or prolactin, which in some women may directly or indirectly result in MD [62]. Tendford et al. [53] underlined that these factors are helpful to guide sports medicine professionals in the evaluation and management of young females at risk for health problems. Therefore, regular and close monitor of young females could minimize MD [63]. Specifically, long training periods and competitions may impair ovarian activity, and this could manifest as luteal phase defects, irregular menstruation, or amenorrhea [64]. These type of disorders are generally accompanied by reduced blood estradiol and progesterone levels during the luteal phase [65]. Consequently, the alterations in hormone levels might lead to either improved or decreased performance at various times throughout the MC [66]. In endurance sports, such as swimming, where muscular strength plays a relevant role, the prevalence of MD ranged between 15% and 82% [67,68]. Furthermore, Schtscherbyna et al. [69] showed that a significant number of swimmers living with triad syndrome reported a prevalence of oligomenorrhea. Muia et al. [70] reported similar data on subclinical and/or clinical triad components in female runners. 

The prevalence of MD spans various competitive disciplines, and disordered eating habits can persist due to the emphasis often placed on body size and/or body composition. Specifically, the relentless pursuit of leaner physiques is frequently regarded as a legitimate performance-enhancing strategy within the sport context and is further reinforced by sociocultural pressures outside the competitive environment [71]. Young girls engage in intense training regimes long before menarche in disciplines such as rhythmic gymnastics, where the measure of athletes’ success is usually strongly influenced by visual appeal and body esthetics [72,73]. Accordingly, the reduction in growth potential due to an inadequate training intensity can cause primary amenorrhea, secondary amenorrhea, or the total absence of a menstrual cycle. 

Numerous studies have confirmed the high prevalence of MD in the field of gymnastics. For example, Klinkowski et al. [74] showed that intensive physical activity undertaken by athletes promotes premenstrual syndrome and MD. Similarly, the existing literature describes a high prevalence of delayed menarches and MD in the population of ballet dancers, reinforcing the association between the late onset of menarche and the presence of amenorrhea [75]. Also, Liu et al. found a high prevalence of primary and secondary amenorrhea among young elite dancers as a consequence of the volume and the intensity of the training sessions [76]. 

MD may not only be related to individual sports, but also team sports; Dusek et al. [49] found that basketball and volleyball players suffered from secondary amenorrhea and, as for previous studies, menarche was significantly delayed in the athletes who engaged in intense physical activities during pre-adolescence. Supporting these findings, Beals et al. [60] examined the menstrual function of volleyball players, noticing that the occurrence of amenorrhea, oligomenorrhea, and irregular MC increased during the competitive season. As previously discussed, high doses of physical exercise may induce MD. For instance, Prather et al. [77] reported similar findings in elite female soccer athletes who showed susceptibility to stress fractures and MD.

In Table 2, the main studies on physical exercise and MD in adolescence are presented.

### 5.2. Impact of Training Program on Menstrual Dysfunction

Several factors such as training type, volume, and intensity may account for the prevalence of variability in MD across different sports [49]. Indeed, every sport can be classified by the percentage of aerobic/anaerobic production of energy [85] or by energy system involvement [86]. Consequently, training sessions aim to enhance performance in accordance with the specific requirements. On the other hand, there are many sources of variability, such as the season, the technical and tactical needs, and the athlete level, considerably varying the training programs.

Female athletes exposed to intensive training and psychological stress, as well as to weight-sensitive and leanness sports, are more prone to develop MD. 

Specifically, a high risk for MD is more evident in disciplines mainly involving endurance or emphasizing weight categories or in those centered on esthetics such as long-distance running, rhythmic gymnastics, and dance. 

The study of Rauh et al. [82] supports this theory, reporting that menstrual irregularities are typical of high school highly specialized distance runners compared to their low-specialized counterparts. Similarly, findings by Czajkowska et al. [80] reported that middle- and long-distance runners suffer from significantly longer menstrual intervals. In addition, the study showed that intensive physical activity during pre-adolescence is a menarche-delaying factor and that participation in competitive sports seems to worsen premenstrual syndrome and premenstrual dysphoric disorder. 

Unfortunately, prospective studies of exercise training in adolescents are limited and, therefore, a direct correlation between the length and frequency of training sessions and MD is not fully elucidated.

Recently, Amoruso et al. [87] did not find any statistical correlation between the length and frequency of training sessions with menstrual irregularity in a sample of 288 female athletes (of them, 73.3% were under 25 years of age). 

Dusek et al. [49] showed that MD was more common in long-distance runners than in short-distance runners, although there were no significant differences in the training load between these two subgroups. The differences in constitution, body weight, and training pattern may explain the variable prevalence [88]; in fact, the training of long-distance runners is characterized by longer, continuous, aerobic exercises of somewhat lower intensity, and they have a slighter muscular structure and lower body weight compared to short-distance runners [88]. 

In female athletes who compete in running disciplines, Passoni et al. [89] reported that the variable of kilometers run per week (for 10 km, OR 1.35) was associated with menstrual irregularities, and the cut-off of 65 km run per week is a good indicator of the presence of an irregular menstrual cycle.

Further studies, starting early after menarche, are necessary to establish real knowledge on the correlation between MD and the intensity, specific type, and amount of training in adolescents.

## 6. Limits

Certainly, this review has some limitations. Firstly, we present a narrative review, which, as noted by Gregory et al. [15], offers a non-systematic overview and analysis of the existing literature on a particular topic. The non-systematic nature of narrative reviews means there are no formally established guidelines for their conduct, potentially introducing biases in selection and often resulting in qualitative syntheses. For instance, we only reviewed articles available on PubMed, which means that relevant studies from other databases and search engines might have been excluded.

Secondly, discussions of MD in athletes have long been around, and one may argue that this is a topic lacking originality. However, studies specifically focused on the adolescent age group are limited and we believe that the absence of specific guidelines for monitoring the menstrual health of adolescents highlights the importance of focusing on this age group. Indeed, physiological variability is more pronounced, requiring even more personalized educational and preventive interventions. 

Additionally, the literature shows reports with small sample sizes and highly heterogeneous populations, which makes it difficult to compare studies adequately. In most cases, the prevalence of MD was reported based on types of exercise. Instead, more prospective studies on exercise training (independently varying the amount, intensity, and rate of change) and energy (independently varying caloric expenditure, caloric intake, and caloric deficit) are necessary to establish a direct correlation between MD and the intensity, specific type, and amount of training in adolescents.

Finally, the reported studies include cases that are not well characterized from a hormonal perspective, underlining the need to conduct comprehensive and multidisciplinary studies in order to fully understand the pathogenetic mechanisms of the association between exercise and MD.

## 7. Conclusions

MD is frequently observed in women participating in physical activity. Several factors should be considered when addressing MD in young athletes. Firstly, some disciplines have a higher prevalence of MD due to high training loads and early introduction to competitive careers. Moreover, low energy intake and a low body mass index can exacerbate existing MD and alter athletes’ menstrual cycles. Lastly, disordered eating behaviors and psychological stress contribute to MD in female athletes. Psychological pressure is often perceived by athletes and may be a contributing factor to MD [32].

Early recognition and management of MD in female athletes are crucial. Although there are currently no specific guidelines for monitoring menstrual status in adolescents within a sports setting, introducing tools for tracking the menstrual cycle, such as online applications [90,91], could be valuable in establishing an athlete’s menstrual profile. By identifying this profile, coaches can assess the impact of menstrual status on training and performance, enabling them to adjust programs as needed. Furthermore, early detection of significant variations in cycle length allows for the timely investigation of potential causes, such as imbalances in training load and energy intake or stress [78]. Then, appropriate strategies can be implemented and evaluated more efficiently.

Regular monitoring of menstrual health and implementing educational programs for female athletes and coaches about risks and prevention strategies are vital. Sports organizations and health professionals should collaborate to create training environments that minimize MD risks by ensuring proper nutrition and balancing training and recovery. Understanding factors contributing to MD in adolescent female athletes can promote awareness, facilitate early diagnosis, and support treatment strategies [25], reducing injury risk and enhancing sports performance. 

## Figures and Tables

**Table 1:** Main causes of primary/secondary amenorrhea and other menstrual abnormalities.

Amenorrhea	
Disorders of Outflow Tract and/or Uterus	Cryptomenorrhea (vaginal atresia/hypoplasia or imperforate hymen) Testicular feminization (Androgen Insensitivity) Asherman’s syndrome Infections
Ovary Disorders	Chromosomal abnormalities (Turner’s syndrome) Gonadal agenesis Resistant ovary syndrome Premature menopause/ovarian insufficiency (autoimmune diseases, viral infection, cytotoxic drugs) Polycystic ovarian syndrome
Anterior Pituitary Disorders	Pituitary tumor causing “hyperprolactinemia” Other causes of hyperprolactinemia Craniopharyngioma Sheehan’s syndrome
Hypothalamus Disorders	Tumors Amenorrhea and anosmia Stress Chronic illness
Other menstrual abnormalities	
Oligomenorrhea/ Polymenorrhea	Endocrine disorders (thyroid dysfunction, hyperandrogenism, hypothalamic–pituitary axis disorder) Polycystic ovarian syndrome Ovarian insufficiency
Heavy Menstrual Bleeding	Immature hypothalamic–pituitary–ovarian axis Coagulation defects Thrombocytopenia or platelet dysfunctions
Abnormal Uterine Bleeding	Structural factors (polyp, adenomyosis, leiomyoma, fibroid, cancer, or hyperplasia) Nonstructural factors (coagulopathy, ovulatory dysfunction, endometrial factors, iatrogenic factors, factors that are not otherwise classified)

**Table 2:** Main studies on physical exercise and menstrual dysfunction (MD).

Author	Sample	Age Range	Sport	Type of Menstrual Dysfunction
Barrack MT, 2008 [58]	93	16.1 ± 0.1 16	Cross-country running	Primary amenorrhea, Secondary amenorrhea, Oligomenorrhea
Barrack MT, 2010 [78]	39	15.7 ± 0.2 15–16	Cross-country running	Primary amenorrhea, Secondary amenorrhea, Oligomenorrhea
Beals KA, 2002 [60]	23	15.8 ± 1.1 14–16	Volleyball	Primary amenorrhea, Oligomenorrhea, Menstrual cycle irregularities
Castelo-Branco, 2006 [75]	38	14.8 ± 1.7 15–17	Ballet	Oligomenorrhea, Menstrual cycle irregularities
de Oliveira Coelho GM, 2013 [54]	24	14.77 ± 2.16 13–17	Tennis	Secondary amenorrhea, Oligomenorrhea, Menstrual cycle irregularities
Coste O, 2011 [68]	18	15.2 ± 1.1 14–16	Swimming	Secondary amenorrhea, Oligomenorrhea
Cristina-Souza G, 2019 [66]	12	16.5 ±1.6 14–18	Track and field	Dysmenorrhea
Czajkowska M, 2019 [79]	45	16.28 ± 0.84 15–16	Rhythmic gymnastics	Secondary amenorrhea, Oligomenorrhea, Hypomenorrhea
Czajkowska M, 2020 [80]	75	18.6 ± 1.9 17–20	Middle- and long-distance running	Secondary amenorrhea, Oligomenorrhea,
Di Cagno A, 2012 [73]	46	17.4 ± 3.0 14–20	Rhythmic gymnasts	Secondary amenorrhea, Menstrual cycle irregularities
Dusek T, 2001 [49]	72	15–19	Basketball, volleyball, ballet, track and field	Primary amenorrhea, Secondary amenorrhea
Egan E, 2003 [81]	37	17.5 ± 3.4 15–21	Figure skating	Secondary amenorrhea, Oligomenorrhea
Felmann JM, 2011 [64]	103	15–17	Track and field	Secondary amenorrhea, Oligomenorrhea
Helge EW, 2002 [62]	6	17.9 ± 1.5 17–19	Artistic gymnastics	Primary amenorrhea, Secondary amenorrhea, Oligomenorrhea
Klentrou P, 2003 [72]	23	14.7 ± 0.4 14–15	Rhythmic gymnastics	Secondary amenorrhea, Oligomenorrhea
Klinkowski N, 2008 [74]	51	15.2 ± 1.8	Rhythmic gymnastics	Secondary amenorrhea
Liu Z, 2024 [76]	131	15.9 ± 1.5	Elite dance	Primary and Secondary amenorrhea
Muia EN, 2016 [70]	56	16.0	Middle-distance running	Primary amenorrhea, Secondary amenorrhea
Prather H, 2016 [77]	220	16.4 ± 4	Soccer	Primary amenorrhea
Rauh MJ, 2014 [63]	89	15.5 ± 1.3	Middle-distance running	Menstrual cycle irregularities
Rauh MJ, 2020 [82]	64	15.6 ± 1.4	Running	Heavy menstrual bleeding or longer breaks between menstrual bleeds
Roupas ND, 2014 [83]	77	18.3 ± 2.6	Rhythmic gymnastics	Primary amenorrhea, Oligomenorrhea
Salbach H, 2007 [71]	50	14.8 ± 2.1	Rhythmic gymnastics	Primary amenorrhea, Secondary amenorrhea
Schtscherbyna A, 2009 [69]	78	16.7 ± 1.2	Swimming	Oligomenorrhea
Tenforde AS, 2015 [84]	91	16.9 ± 1.3	Middle-distance running	Primary amenorrhea, Secondary amenorrhea
Thompson SH, 2007 [59]	300	19.64 ± 1.56	Cross-country running	Secondary amenorrhea, Oligomenorrhea
Tsukahara Y, 2021 [61]	91	18.10 ± 0.37	Track and field	Secondary amenorrhea, Oligomenorrhea
